# Puerperal Convulsions

**Published:** 1890-10

**Authors:** Charles P. Beaman

**Affiliations:** Chattanooga, Tenn.


### PUERPERAL CONVULSIONS.

CHARLES P. BEAMAN, M. D., CHATTANOOGA, TENN.

August 12th, was hastily summoned at four P. M. to see a Mrs. W., who the messenger said was "taken bad with fits."

Upon my arrival the patient had just recovered from her second convulsion, during which she had bitten the tongue, it being greatly swollen, rendering intelligible speech impossible. She was about to be confined with her second child, and the nurse said had been complaining of considerable pain about the abdomen for a day or two, but "This morning had been took bad with a terrible pain in her head, and I hain't heared her say nothing about those stomach pains since." This was all I was able to learn about the case—the patient could tell me nothing; so I concluded to watch her through another convulsion before prescribing, giving a placebo at once for the benefit of the neighbors. I had not long to wait; presently there was twitching of the hands and arms, slight at first, but gradually becoming more decided; then a similar twitching of the muscles of the face; then of the lower limbs; the eyelids opened and shut spasmodically, when suddenly the whole body became rigid for an instant, then twitched and jerked so violently that the room fairly shook, fingers clenched, but not upon the thumbs, face horribly swollen and almost black, eyes wide open, pupils dilated and insensible to light, tongue partially protruded and blue, froth and bloody saliva running from mouth; neck and parotid glands greatly swollen; *hot perspiration* all over the body. Presently there was a momentary cessation of breathing followed by quick, short gasps, then labored respiration; short inspiration, and long, loud, blowing expiration.

This condition, after thirty-five minutes, lapsed into a heavy, stertorous sleep.

An examination per vaginam during this attack revealed the fact that the uterus did not participate in any degree in these muscular spasms, but remained perfectly passive, the os soft and dilated about the size of a half dollar. I could see nothing but *Opium* for this case, so, dissolving Op.$^{cc}$ in a little water, gave two teaspoonfuls every ten minutes until she had three doses. In about an hour her restlessness at intervals led me to place my hand upon the abdomen, when I discovered there were weak but tangible uterine contractions.

In five hours from last spasm another one came on with same symptoms as before, but less violent, and lasted but eighteen minutes, after which uterine contractions continued about as before.

In three hours another spasm, rather more severe than the last, and of twenty-five minutes duration.

I now administered *Opium*$^{dmm}$ (Swan), one dose dry. In one hour the patient was breathing quite naturally, would awaken during each pain, which were now more frequent and severe; she seemed perfectly conscious and would drink water or a little oatmeal gruel with apparent relish. Soon she attempted to get out of bed as each pain came on to use the vessel; this symptom became such a marked feature, and as the pain seemed lacking in expulsive force, I gave *Nux-vom.*$^{cc}$, the effect of which was speedily apparent; in another hour she gave birth to a fine big boy, and the labor terminated normally in every respect. The patient has since made a good recovery, no more medicine being given except a dose of Arnica$^{cc}$ after confinement to relieve the intense soreness.

She says that she has not the slightest recollection of anything that occurred during her sickness—it is all a blank to her.

Can the "*mongrels*" or "*regulars*" show such quick and positive results with their methods of treatment? I think not.

After the second dose of Opium scarcely a symptom appeared that caused me any apprehension.

